# Dr. Andrews, on Accommodations at Madeira

**Published:** 1808-01

**Authors:** M. W. Andrews

**Affiliations:** Madeira


### *To the Editors of the Medical and Physical Journal.*

Gentlemen,

In the Medical Journal for April 1807, No. 98, page 391, I met with a paragraph, under the article "Intelligence," relative to the island of Madeira; which, as it is calculated to mislead invalids and medical men in England, I feel it my duty, as a professional man, and a practitioner on the island, to correct.

The representation, that "the difficulty so long complained of, and so universally lamented, the want of proper accommodations for strangers being now removed;" "and that houses are now erected for families in sufficient number to produce a useful competition," is by no means true. That strangers arriving here, are now more easily and more comfortably accommodated than they could be some years ago, is certainly true; but that the accommodations are such as to remove every difficulty to the invalid, who is sent here under circumstances requiring not only lodgings, but also several other comforts, equally necessary for his situation, is by no means the case; insomuch, that medical men, advising their patients to resort to this island, for the benefit which the salubrity of the air is likely to afford, ought at the same time to point out to them the necessity of providing themselves with such comforts as their situation may render it probable for them to require; and if no part of their family can accompany them, a trusty servant should undoubtedly be procured.—With respect to families, the same circumstances should also be attended to.

I am, &c.

